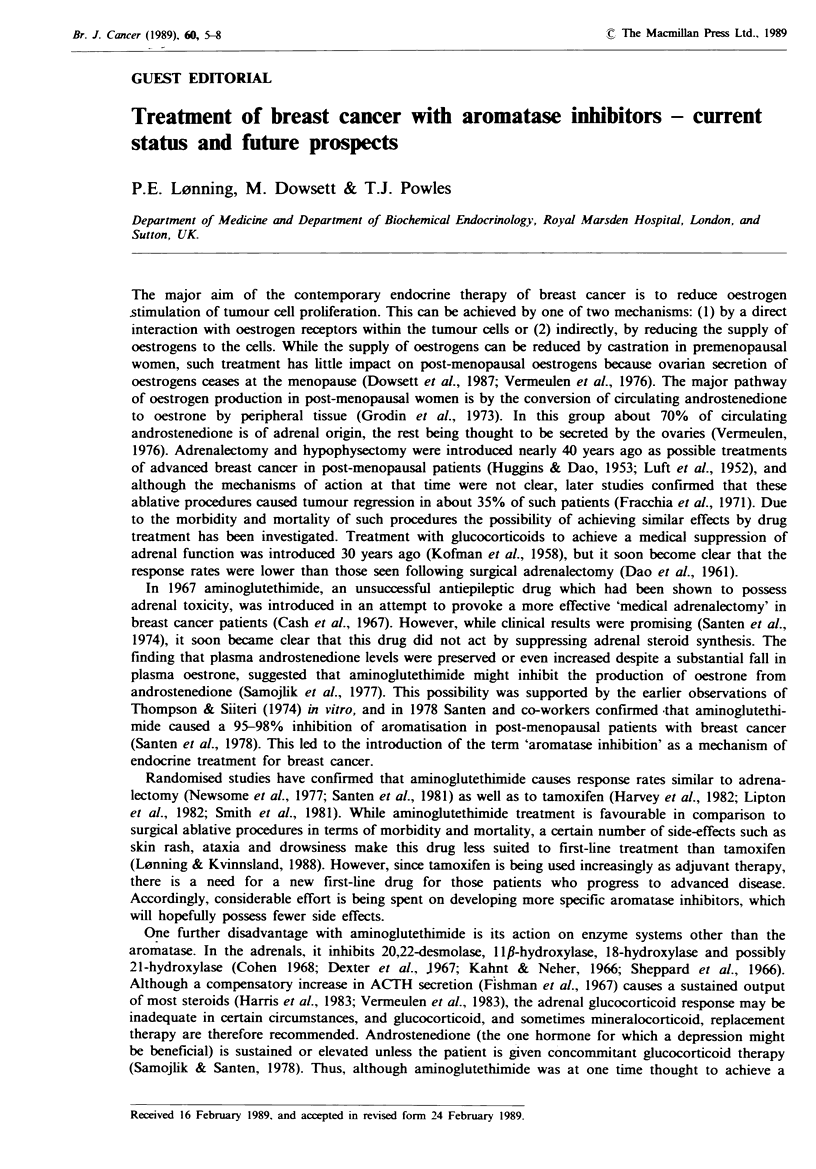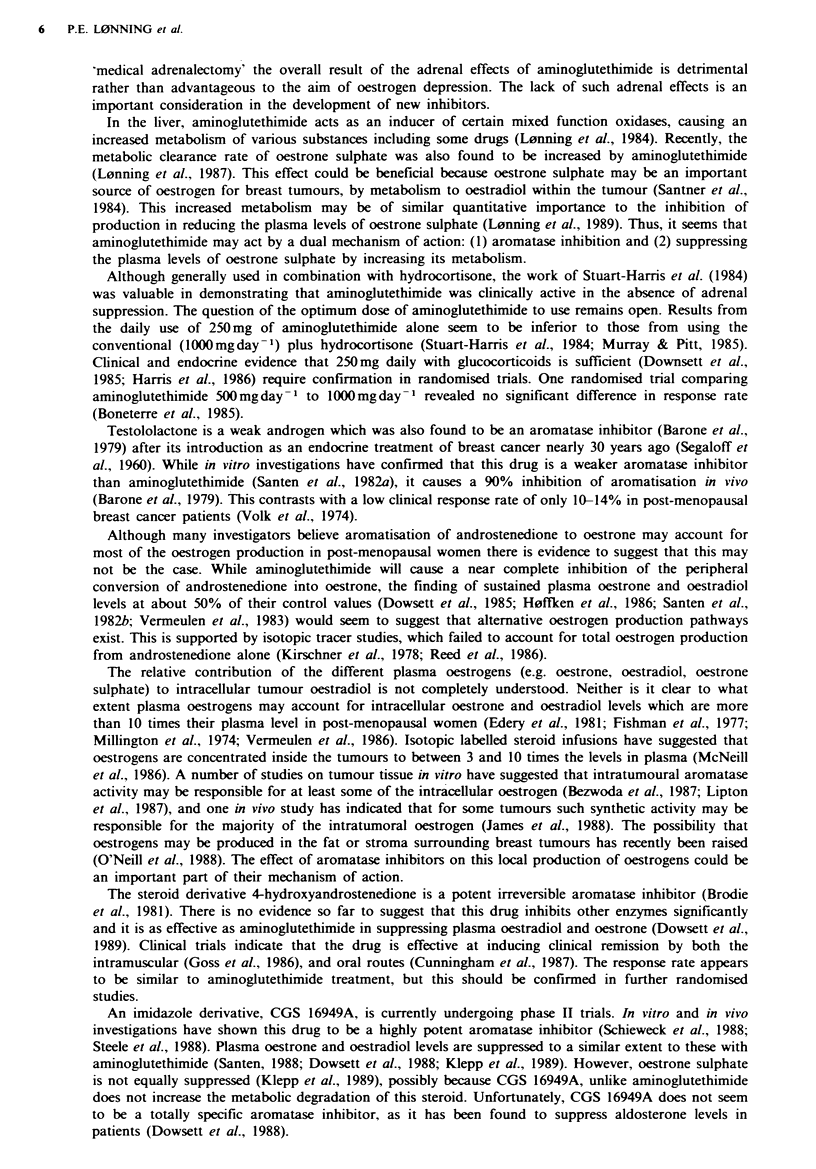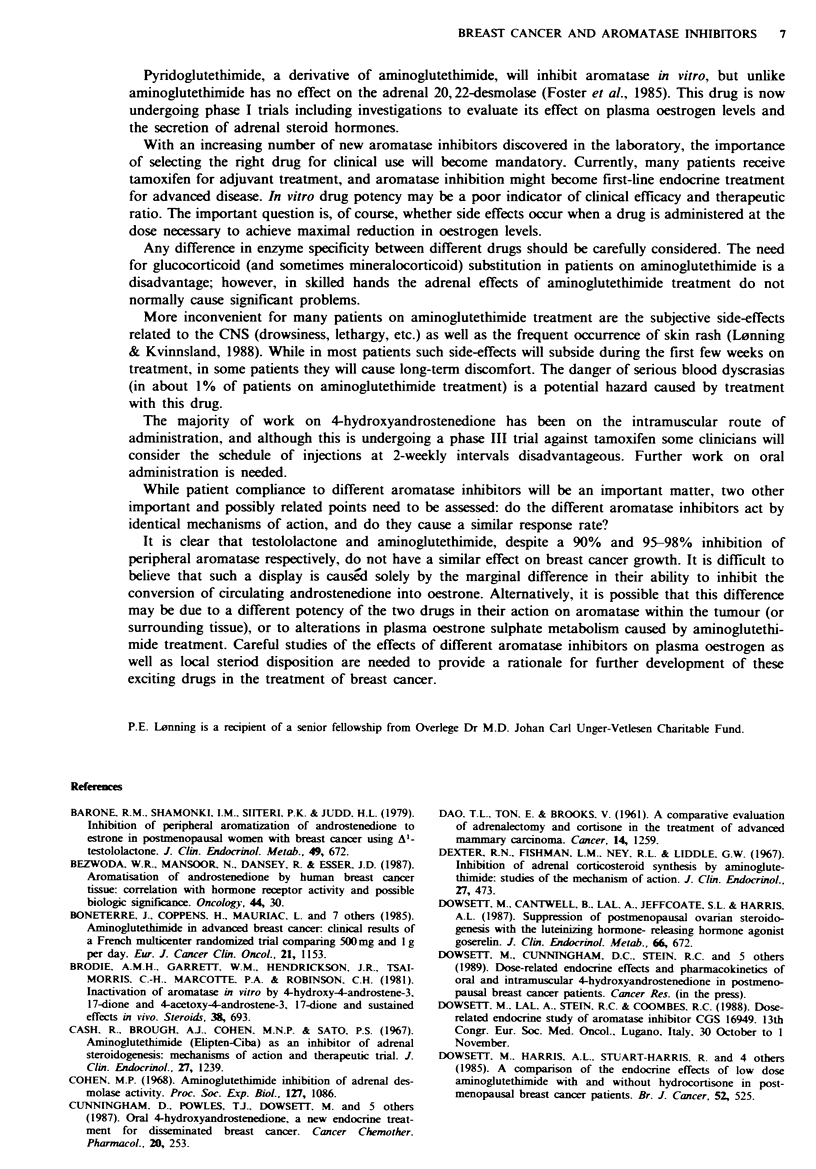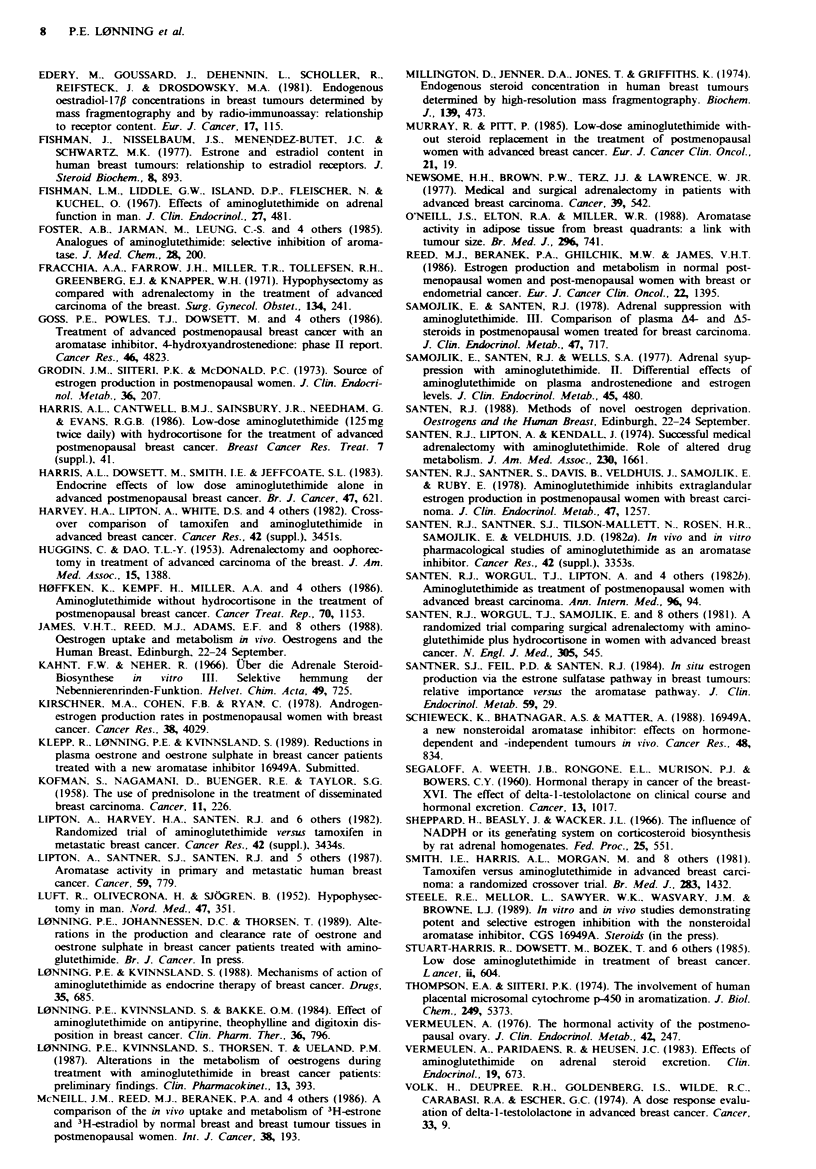# Treatment of breast cancer with aromatase inhibitors--current status and future prospects.

**DOI:** 10.1038/bjc.1989.208

**Published:** 1989-07

**Authors:** P. E. LÃ¸nning, M. Dowsett, T. J. Powles

**Affiliations:** Department of Medicine, Royal Marsden Hospital, London.


Br. J. Cancer (1989), 60, 5–8 © The Macmillan Press Ltd., 1989

GUEST EDITORIAL

### Treatment of breast cancer with aromatase inhibitors – current status and future prospects

P.E. Lønning, M. Dowsett & T.J. Powles

*Department of Medicine and Department of Biochemical Endocrinology, Royal Marsden Hospital, London, and Sutton, UK.*

The major aim of the contemporary endocrine therapy of breast cancer is to reduce oestrogen stimulation of tumour cell proliferation. This can be achieved by one of two mechanisms: (1) by a direct interaction with oestrogen receptors within the tumour cells or (2) indirectly, by reducing the supply of oestrogens to the cells. While the supply of oestrogens can be reduced by castration in premenopausal women, such treatment has little impact on post-menopausal oestrogens because ovarian secretion of oestrogens ceases at the menopause (Dowsett *et al.*, 1987; Vermeulen *et al.*, 1976). The major pathway of oestrogen production in post-menopausal women is by the conversion of circulating androstenedione to oestrone by peripheral tissue (Grodin *et al.*, 1973). In this group about 70% of circulating androstenedione is of adrenal origin, the rest being thought to be secreted by the ovaries (Vermeulen, 1976). Adrenalectomy and hypophysectomy were introduced nearly 40 years ago as possible treatments of advanced breast cancer in post-menopausal patients (Huggins & Dao, 1953; Luft *et al.*, 1952), and although the mechanisms of action at that time were not clear, later studies confirmed that these ablative procedures caused tumour regression in about 35% of such patients (Fracchia *et al.*, 1971). Due to the morbidity and mortality of such procedures the possibility of achieving similar effects by drug treatment has been investigated. Treatment with glucocorticoids to achieve a medical suppression of adrenal function was introduced 30 years ago (Kofman *et al.*, 1958), but it soon become clear that the response rates were lower than those seen following surgical adrenalectomy (Dao *et al.*, 1961).

In 1967 aminoglutethimide, an unsuccessful antiepileptic drug which had been shown to possess adrenal toxicity, was introduced in an attempt to provoke a more effective 'medical adrenalectomy' in breast cancer patients (Cash *et al.*, 1967). However, while clinical results were promising (Santen *et al.*, 1974), it soon became clear that this drug did not act by suppressing adrenal steroid synthesis. The finding that plasma androstenedione levels were preserved or even increased despite a substantial fall in plasma oestrone, suggested that aminoglutethimide might inhibit the production of oestrone from androstenedione (Samojlik *et al.*, 1977). This possibility was supported by the earlier observations of Thompson & Siiteri (1974) *in vitro*, and in 1978 Santen and co-workers confirmed that aminoglutethimide caused a 95–98% inhibition of aromatisation in post-menopausal patients with breast cancer (Santen *et al.*, 1978). This led to the introduction of the term 'aromatase inhibition' as a mechanism of endocrine treatment for breast cancer.

Randomised studies have confirmed that aminoglutethimide causes response rates similar to adrenalectomy (Newsome *et al.*, 1977; Santen *et al.*, 1981) as well as to tamoxifen (Harvey *et al.*, 1982; Lipton *et al.*, 1982; Smith *et al.*, 1981). While aminoglutethimide treatment is favourable in comparison to surgical ablative procedures in terms of morbidity and mortality, a certain number of side-effects such as skin rash, ataxia and drowsiness make this drug less suited to first-line treatment than tamoxifen (Lønning & Kvinnsland, 1988). However, since tamoxifen is being used increasingly as adjuvant therapy, there is a need for a new first-line drug for those patients who progress to advanced disease. Accordingly, considerable effort is being spent on developing more specific aromatase inhibitors, which will hopefully possess fewer side effects.

One further disadvantage with aminoglutethimide is its action on enzyme systems other than the aromatase. In the adrenals, it inhibits 20,22-desmolase, 11$\beta$-hydroxylase, 18-hydroxylase and possibly 21-hydroxylase (Cohen 1968; Dexter *et al.*, 1967; Kahnt & Neher, 1966; Sheppard *et al.*, 1966). Although a compensatory increase in ACTH secretion (Fishman *et al.*, 1967) causes a sustained output of most steroids (Harris *et al.*, 1983; Vermeulen *et al.*, 1983), the adrenal glucocorticoid response may be inadequate in certain circumstances, and glucocorticoid, and sometimes mineralocorticoid, replacement therapy are therefore recommended. Androstenedione (the one hormone for which a depression might be beneficial) is sustained or elevated unless the patient is given concommitant glucocorticoid therapy (Samojlik & Santen, 1978). Thus, although aminoglutethimide was at one time thought to achieve a

Received 16 February 1989, and accepted in revised form 24 February 1989.

'medical adrenalectomy' the overall result of the adrenal effects of aminoglutethimide is detrimental rather than advantageous to the aim of oestrogen depression. The lack of such adrenal effects is an important consideration in the development of new inhibitors.

In the liver, aminoglutethimide acts as an inducer of certain mixed function oxidases, causing an increased metabolism of various substances including some drugs (Lønning *et al.*, 1984). Recently, the metabolic clearance rate of oestrone sulphate was also found to be increased by aminoglutethimide (Lønning *et al.*, 1987). This effect could be beneficial because oestrone sulphate may be an important source of oestrogen for breast tumours, by metabolism to oestradiol within the tumour (Santner *et al.*, 1984). This increased metabolism may be of similar quantitative importance to the inhibition of production in reducing the plasma levels of oestrone sulphate (Lønning *et al.*, 1989). Thus, it seems that aminoglutethimide may act by a dual mechanism of action: (1) aromatase inhibition and (2) suppressing the plasma levels of oestrone sulphate by increasing its metabolism.

Although generally used in combination with hydrocortisone, the work of Stuart-Harris *et al.* (1984) was valuable in demonstrating that aminoglutethimide was clinically active in the absence of adrenal suppression. The question of the optimum dose of aminoglutethimide to use remains open. Results from the daily use of 250 mg of aminoglutethimide alone seem to be inferior to those from using the conventional (1000 mg day$^{-1}$) plus hydrocortisone (Stuart-Harris *et al.*, 1984; Murray & Pitt, 1985). Clinical and endocrine evidence that 250 mg daily with glucocorticoids is sufficient (Downsett *et al.*, 1985; Harris *et al.*, 1986) require confirmation in randomised trials. One randomised trial comparing aminoglutethimide 500 mg day$^{-1}$ to 1000 mg day$^{-1}$ revealed no significant difference in response rate (Boneterre *et al.*, 1985).

Testololactone is a weak androgen which was also found to be an aromatase inhibitor (Barone *et al.*, 1979) after its introduction as an endocrine treatment of breast cancer nearly 30 years ago (Segaloff *et al.*, 1960). While *in vitro* investigations have confirmed that this drug is a weaker aromatase inhibitor than aminoglutethimide (Santen *et al.*, 1982*a*), it causes a 90% inhibition of aromatisation *in vivo* (Barone *et al.*, 1979). This contrasts with a low clinical response rate of only 10–14% in post-menopausal breast cancer patients (Volk *et al.*, 1974).

Although many investigators believe aromatisation of androstenedione to oestrone may account for most of the oestrogen production in post-menopausal women there is evidence to suggest that this may not be the case. While aminoglutethimide will cause a near complete inhibition of the peripheral conversion of androstenedione into oestrone, the finding of sustained plasma oestrone and oestradiol levels at about 50% of their control values (Dowsett *et al.*, 1985; Høffken *et al.*, 1986; Santen *et al.*, 1982*b*; Vermeulen *et al.*, 1983) would seem to suggest that alternative oestrogen production pathways exist. This is supported by isotopic tracer studies, which failed to account for total oestrogen production from androstenedione alone (Kirschner *et al.*, 1978; Reed *et al.*, 1986).

The relative contribution of the different plasma oestrogens (e.g. oestrone, oestradiol, oestrone sulphate) to intracellular tumour oestradiol is not completely understood. Neither is it clear to what extent plasma oestrogens may account for intracellular oestrone and oestradiol levels which are more than 10 times their plasma level in post-menopausal women (Edery *et al.*, 1981; Fishman *et al.*, 1977; Millington *et al.*, 1974; Vermeulen *et al.*, 1986). Isotopic labelled steroid infusions have suggested that oestrogens are concentrated inside the tumours to between 3 and 10 times the levels in plasma (McNeill *et al.*, 1986). A number of studies on tumour tissue *in vitro* have suggested that intratumoural aromatase activity may be responsible for at least some of the intracellular oestrogen (Bezwoda *et al.*, 1987; Lipton *et al.*, 1987), and one *in vivo* study has indicated that for some tumours such synthetic activity may be responsible for the majority of the intratumoral oestrogen (James *et al.*, 1988). The possibility that oestrogens may be produced in the fat or stroma surrounding breast tumours has recently been raised (O'Neill *et al.*, 1988). The effect of aromatase inhibitors on this local production of oestrogens could be an important part of their mechanism of action.

The steroid derivative 4-hydroxyandrostenedione is a potent irreversible aromatase inhibitor (Brodie *et al.*, 1981). There is no evidence so far to suggest that this drug inhibits other enzymes significantly and it is as effective as aminoglutethimide in suppressing plasma oestradiol and oestrone (Dowsett *et al.*, 1989). Clinical trials indicate that the drug is effective at inducing clinical remission by both the intramuscular (Goss *et al.*, 1986), and oral routes (Cunningham *et al.*, 1987). The response rate appears to be similar to aminoglutethimide treatment, but this should be confirmed in further randomised studies.

An imidazole derivative, CGS 16949A, is currently undergoing phase II trials. *In vitro* and *in vivo* investigations have shown this drug to be a highly potent aromatase inhibitor (Schieweck *et al.*, 1988; Steele *et al.*, 1988). Plasma oestrone and oestradiol levels are suppressed to a similar extent to these with aminoglutethimide (Santen, 1988; Dowsett *et al.*, 1988; Klepp *et al.*, 1989). However, oestrone sulphate is not equally suppressed (Klepp *et al.*, 1989), possibly because CGS 16949A, unlike aminoglutethimide does not increase the metabolic degradation of this steroid. Unfortunately, CGS 16949A does not seem to be a totally specific aromatase inhibitor, as it has been found to suppress aldosterone levels in patients (Dowsett *et al.*, 1988).

Pyridoglutethimide, a derivative of aminoglutethimide, will inhibit aromatase *in vitro*, but unlike aminoglutethimide has no effect on the adrenal 20,22-desmolase (Foster *et al.*, 1985). This drug is now undergoing phase I trials including investigations to evaluate its effect on plasma oestrogen levels and the secretion of adrenal steroid hormones.

With an increasing number of new aromatase inhibitors discovered in the laboratory, the importance of selecting the right drug for clinical use will become mandatory. Currently, many patients receive tamoxifen for adjuvant treatment, and aromatase inhibition might become first-line endocrine treatment for advanced disease. *In vitro* drug potency may be a poor indicator of clinical efficacy and therapeutic ratio. The important question is, of course, whether side effects occur when a drug is administered at the dose necessary to achieve maximal reduction in oestrogen levels.

Any difference in enzyme specificity between different drugs should be carefully considered. The need for glucocorticoid (and sometimes mineralocorticoid) substitution in patients on aminoglutethimide is a disadvantage; however, in skilled hands the adrenal effects of aminoglutethimide treatment do not normally cause significant problems.

More inconvenient for many patients on aminoglutethimide treatment are the subjective side-effects related to the CNS (drowsiness, lethargy, etc.) as well as the frequent occurrence of skin rash (Lønning & Kvinnsland, 1988). While in most patients such side-effects will subside during the first few weeks on treatment, in some patients they will cause long-term discomfort. The danger of serious blood dyscrasias (in about 1% of patients on aminoglutethimide treatment) is a potential hazard caused by treatment with this drug.

The majority of work on 4-hydroxyandrostenedione has been on the intramuscular route of administration, and although this is undergoing a phase III trial against tamoxifen some clinicians will consider the schedule of injections at 2-weekly intervals disadvantageous. Further work on oral administration is needed.

While patient compliance to different aromatase inhibitors will be an important matter, two other important and possibly related points need to be assessed: do the different aromatase inhibitors act by identical mechanisms of action, and do they cause a similar response rate?

It is clear that testololactone and aminoglutethimide, despite a 90% and 95–98% inhibition of peripheral aromatase respectively, do not have a similar effect on breast cancer growth. It is difficult to believe that such a display is caused solely by the marginal difference in their ability to inhibit the conversion of circulating androstenedione into oestrone. Alternatively, it is possible that this difference may be due to a different potency of the two drugs in their action on aromatase within the tumour (or surrounding tissue), or to alterations in plasma oestrone sulphate metabolism caused by aminoglutethimide treatment. Careful studies of the effects of different aromatase inhibitors on plasma oestrogen as well as local steriod disposition are needed to provide a rationale for further development of these exciting drugs in the treatment of breast cancer.

P.E. Lønning is a recipient of a senior fellowship from Overlege Dr M.D. Johan Carl Unger-Vetlesen Charitable Fund.

### References

BARONE, R.M., SHAMONKI, I.M., SIITERI, P.K. & JUDD, H.L. (1979). Inhibition of peripheral aromatization of androstenedione to estrone in postmenopausal women with breast cancer using $\Delta^1$-testololactone. *J. Clin. Endocrinol. Metab.*, **49**, 672.

BEZWODA, W.R., MANSOOR, N., DANSEY, R. & ESSER, J.D. (1987). Aromatisation of androstenedione by human breast cancer tissue: correlation with hormone receptor activity and possible biologic significance. *Oncology*, **44**, 30.

BONETERRE, J., COPPENS, H., MAURIAC, L. and 7 others (1985). Aminoglutethimide in advanced breast cancer: clinical results of a French multicenter randomized trial comparing 500 mg and 1 g per day. *Eur. J. Cancer Clin. Oncol.*, **21**, 1153.

BRODIE, A.M.H., GARRETT, W.M., HENDRICKSON, J.R., TSAI-MORRIS, C.-H., MARCOTTE, P.A. & ROBINSON, C.H. (1981). Inactivation of aromatase *in vitro* by 4-hydroxy-4-androstene-3, 17-dione and 4-acetoxy-4-androstene-3, 17-dione and sustained effects *in vivo*. *Steroids*, **38**, 693.

CASH, R., BROUGH, A.J., COHEN, M.N.P. & SATO, P.S. (1967). Aminoglutethimide (Elipten-Ciba) as an inhibitor of adrenal steroidogenesis: mechanisms of action and therapeutic trial. *J. Clin. Endocrinol.*, **27**, 1239.

COHEN, M.P. (1968). Aminoglutethimide inhibition of adrenal desmolase activity. *Proc. Soc. Exp. Biol.*, **127**, 1086.

CUNNINGHAM, D., POWLES, T.J., DOWSETT, M. and 5 others (1987). Oral 4-hydroxyandrostenedione, a new endocrine treatment for disseminated breast cancer. *Cancer Chemother. Pharmacol.*, **20**, 253.

DAO, T.L., TON, E. & BROOKS, V. (1961). A comparative evaluation of adrenalectomy and cortisone in the treatment of advanced mammary carcinoma. *Cancer*, **14**, 1259.

DEXTER, R.N., FISHMAN, L.M., NEY, R.L. & LIDDLE, G.W. (1967). Inhibition of adrenal corticosteroid synthesis by aminoglutethimide: studies of the mechanism of action. *J. Clin. Endocrinol.*, **27**, 473.

DOWSETT, M., CANTWELL, B., LAL, A., JEFFCOATE, S.L. & HARRIS, A.L. (1987). Suppression of postmenopausal ovarian steroidogenesis with the luteinizing hormone- releasing hormone agonist goserelin. *J. Clin. Endocrinol. Metab.*, **66**, 672.

DOWSETT, M., CUNNINGHAM, D.C., STEIN, R.C. and 5 others (1989). Dose-related endocrine effects and pharmacokinetics of oral and intramuscular 4-hydroxyandrostenedione in postmenopausal breast cancer patients. *Cancer Res.* (in the press).

DOWSETT, M., LAL, A., STEIN, R.C. & COOMBES, R.C. (1988). Dose-related endocrine study of aromatase inhibitor CGS 16949. 13th Congr. Eur. Soc. Med. Oncol., Lugano, Italy, 30 October to 1 November.

DOWSETT, M., HARRIS, A.L., STUART-HARRIS, R. and 4 others (1985). A comparison of the endocrine effects of low dose aminoglutethimide with and without hydrocortisone in postmenopausal breast cancer patients. *Br. J. Cancer*, **52**, 525.

EDERY, M., GOUSSARD, J., DEHENNIN, L., SCHOLLER, R., REIFSTECK, J. & DROSDOWSKY, M.A. (1981). Endogenous oestradiol-17β concentrations in breast tumours determined by mass fragmentography and by radio-immunoassay: relationship to receptor content. *Eur. J. Cancer*, **17**, 115.

FISHMAN, J., NISSELBAUM, J.S., MENENDEZ-BUTET, J.C. & SCHWARTZ, M.K. (1977). Estrone and estradiol content in human breast tumours: relationship to estradiol receptors. *J. Steroid Biochem.*, **8**, 893.

FISHMAN, L.M., LIDDLE, G.W., ISLAND, D.P., FLEISCHER, N. & KUCHEL, O. (1967). Effects of aminoglutethimide on adrenal function in man. *J. Clin. Endocrinol.*, **27**, 481.

FOSTER, A.B., JARMAN, M., LEUNG, C.-S. and 4 others (1985). Analogues of aminoglutethimide: selective inhibition of aromatase. *J. Med. Chem.*, **28**, 200.

FRACCHIA, A.A., FARROW, J.H., MILLER, T.R., TOLLEFSEN, R.H., GREENBERG, E.J. & KNAPPER, W.H. (1971). Hypophysectomy as compared with adrenalectomy in the treatment of advanced carcinoma of the breast. *Surg. Gynecol. Obstet.*, **134**, 241.

GOSS, P.E., POWLES, T.J., DOWSETT, M. and 4 others (1986). Treatment of advanced postmenopausal breast cancer with an aromatase inhibitor, 4-hydroxyandrostenedione: phase II report. *Cancer Res.*, **46**, 4823.

GRODIN, J.M., SIITERI, P.K. & McDONALD, P.C. (1973). Source of estrogen production in postmenopausal women. *J. Clin. Endocrinol. Metab.*, **36**, 207.

HARRIS, A.L., CANTWELL, B.M.J., SAINSBURY, J.R., NEEDHAM, G. & EVANS, R.G.B. (1986). Low-dose aminoglutethimide (125 mg twice daily) with hydrocortisone for the treatment of advanced postmenopausal breast cancer. *Breast Cancer Res. Treat.* **7** (suppl.), 41.

HARRIS, A.L., DOWSETT, M., SMITH, I.E. & JEFFCOATE, S.L. (1983). Endocrine effects of low dose aminoglutethimide alone in advanced postmenopausal breast cancer. *Br. J. Cancer*, **47**, 621.

HARVEY, H.A., LIPTON, A., WHITE, D.S. and 4 others (1982). Crossover comparison of tamoxifen and aminoglutethimide in advanced breast cancer. *Cancer Res.*, **42** (suppl.), 3451s.

HUGGINS, C. & DAO, T.L.-Y. (1953). Adrenalectomy and oophorectomy in treatment of advanced carcinoma of the breast. *J. Am. Med. Assoc.*, **15**, 1388.

HØFFKEN, K., KEMPF, H., MILLER, A.A. and 4 others (1986). Aminoglutethimide without hydrocortisone in the treatment of postmenopausal breast cancer. *Cancer Treat. Rep.*, **70**, 1153.

JAMES, V.H.T., REED, M.J., ADAMS, E.F. and 8 others (1988). Oestrogen uptake and metabolism *in vivo*. Oestrogens and the Human Breast, Edinburgh, 22–24 September.

KAHNT, F.W. & NEHER, R. (1966). Über die Adrenale Steroid-Biosynthese *in vitro* III. Selektive hemmung der Nebennierenrinden-Funktion. *Helvet. Chim. Acta*, **49**, 725.

KIRSCHNER, M.A., COHEN, F.B. & RYAN, C. (1978). Androgen-estrogen production rates in postmenopausal women with breast cancer. *Cancer Res.*, **38**, 4029.

KLEPP, R., LØNNING, P.E. & KVINNSLAND, S. (1989). Reductions in plasma oestrone and oestrone sulphate in breast cancer patients treated with a new aromatase inhibitor 16949A. Submitted.

KOFMAN, S., NAGAMANI, D., BUENGER, R.E. & TAYLOR, S.G. (1958). The use of prednisolone in the treatment of disseminated breast carcinoma. *Cancer*, **11**, 226.

LIPTON, A., HARVEY, H.A., SANTEN, R.J. and 6 others (1982). Randomized trial of aminoglutethimide *versus* tamoxifen in metastatic breast cancer. *Cancer Res.*, **42** (suppl.), 3434s.

LIPTON, A., SANTNER, S.J., SANTEN, R.J. and 5 others (1987). Aromatase activity in primary and metastatic human breast cancer. *Cancer*, **59**, 779.

LUFT, R., OLIVECRONA, H. & SJÖGREN, B. (1952). Hypophysectomy in man. *Nord. Med.*, **47**, 351.

LØNNING, P.E., JOHANNESSEN, D.C. & THORSEN, T. (1989). Alterations in the production and clearance rate of oestrone and oestrone sulphate in breast cancer patients treated with aminoglutethimide. *Br. J. Cancer*. In press.

LØNNING, P.E. & KVINNSLAND, S. (1988). Mechanisms of action of aminoglutethimide as endocrine therapy of breast cancer. *Drugs*, **35**, 685.

LØNNING, P.E., KVINNSLAND, S. & BAKKE, O.M. (1984). Effect of aminoglutethimide on antipyrine, theophylline and digitoxin disposition in breast cancer. *Clin. Pharm. Ther.*, **36**, 796.

LØNNING, P.E., KVINNSLAND, S., THORSEN, T. & UELAND, P.M. (1987). Alterations in the metabolism of oestrogens during treatment with aminoglutethimide in breast cancer patients: preliminary findings. *Clin. Pharmacokinet.*, **13**, 393.

McNEILL, J.M., REED, M.J., BERANEK, P.A. and 4 others (1986). A comparison of the *in vivo* uptake and metabolism of $^{3}$H-estrone and $^{3}$H-estradiol by normal breast and breast tumour tissues in postmenopausal women. *Int. J. Cancer*, **38**, 193.

MILLINGTON, D., JENNER, D.A., JONES, T. & GRIFFITHS, K. (1974). Endogenous steroid concentration in human breast tumours determined by high-resolution mass fragmentography. *Biochem. J.*, **139**, 473.

MURRAY, R. & PITT, P. (1985). Low-dose aminoglutethimide without steroid replacement in the treatment of postmenopausal women with advanced breast cancer. *Eur. J. Cancer Clin. Oncol.*, **21**, 19.

NEWSOME, H.H., BROWN, P.W., TERZ, J.J. & LAWRENCE, W. JR. (1977). Medical and surgical adrenalectomy in patients with advanced breast carcinoma. *Cancer*, **39**, 542.

O'NEILL, J.S., ELTON, R.A. & MILLER, W.R. (1988). Aromatase activity in adipose tissue from breast quadrants: a link with tumour size. *Br. Med. J.*, **296**, 741.

REED, M.J., BERANEK, P.A., GHILCHIK, M.W. & JAMES, V.H.T. (1986). Estrogen production and metabolism in normal postmenopausal women and post-menopausal women with breast or endometrial cancer. *Eur. J. Cancer Clin. Oncol.*, **22**, 1395.

SAMOJLIK, E. & SANTEN, R.J. (1978). Adrenal suppression with aminoglutethimide. III. Comparison of plasma Δ4- and Δ5-steroids in postmenopausal women treated for breast carcinoma. *J. Clin. Endocrinol. Metab.*, **47**, 717.

SAMOJLIK, E., SANTEN, R.J. & WELLS, S.A. (1977). Adrenal syuppression with aminoglutethimide. II. Differential effects of aminoglutethimide on plasma androstenedione and estrogen levels. *J. Clin. Endocrinol. Metab.*, **45**, 480.

SANTEN, R.J. (1988). Methods of novel oestrogen deprivation. *Oestrogens and the Human Breast*, Edinburgh, 22–24 September.

SANTEN, R.J., LIPTON, A. & KENDALL, J. (1974). Successful medical adrenalectomy with aminoglutethimide. Role of altered drug metabolism. *J. Am. Med. Assoc.*, **230**, 1661.

SANTEN, R.J., SANTNER, S., DAVIS, B., VELDHUIS, J., SAMOJLIK, E. & RUBY, E. (1978). Aminoglutethimide inhibits extraglandular estrogen production in postmenopausal women with breast carcinoma. *J. Clin. Endocrinol. Metab.*, **47**, 1257.

SANTEN, R.J., SANTNER, S.J., TILSON-MALLETT, N., ROSEN, H.R., SAMOJLIK, E. & VELDHUIS, J.D. (1982*a*). *In vivo* and *in vitro* pharmacological studies of aminoglutethimide as an aromatase inhibitor. *Cancer Res.*, **42** (suppl.), 3353s.

SANTEN, R.J., WORGUL, T.J., LIPTON, A. and 4 others (1982*b*). Aminoglutethimide as treatment of postmenopausal women with advanced breast carcinoma. *Ann. Intern. Med.*, **96**, 94.

SANTEN, R.J., WORGUL, T.J., SAMOJLIK, E. and 8 others (1981). A randomized trial comparing surgical adrenalectomy with aminoglutethimide plus hydrocortisone in women with advanced breast cancer. *N. Engl. J. Med.*, **305**, 545.

SANTNER, S.J., FEIL, P.D. & SANTEN, R.J. (1984). *In situ* estrogen production via the estrone sulfatase pathway in breast tumours: relative importance *versus* the aromatase pathway. *J. Clin. Endocrinol. Metab.* **59**, 29.

SCHIEWECK, K., BHATNAGAR, A.S. & MATTER, A. (1988). 16949A, a new nonsteroidal aromatase inhibitor: effects on hormone-dependent and -independent tumours *in vivo*. *Cancer Res.*, **48**, 834.

SEGALOFF, A. WEETH, J.B., RONGONE, E.L., MURISON, P.J. & BOWERS, C.Y. (1960). Hormonal therapy in cancer of the breast-XVI. The effect of delta-1-testololactone on clinical course and hormonal excretion. *Cancer*, **13**, 1017.

SHEPPARD, H., BEASLY, J. & WACKER, J.L. (1966). The influence of NADPH or its generating system on corticosteroid biosynthesis by rat adrenal homogenates. *Fed. Proc.*, **25**, 551.

SMITH, I.E., HARRIS, A.L., MORGAN, M. and 8 others (1981). Tamoxifen versus aminoglutethimide in advanced breast carcinoma: a randomized crossover trial. *Br. Med. J.*, **283**, 1432.

STEELE, R.E., MELLOR, L., SAWYER, W.K., WASVARY, J.M. & BROWNE, L.J. (1989). *In vitro* and *in vivo* studies demonstrating potent and selective estrogen inhibition with the nonsteroidal aromatase inhibitor, CGS 16949A. *Steroids* (in the press).

STUART-HARRIS, R., DOWSETT, M., BOZEK, T. and 6 others (1985). Low dose aminoglutethimide in treatment of breast cancer. *Lancet*, **ii**, 604.

THOMPSON, E.A. & SIITERI, P.K. (1974). The involvement of human placental microsomal cytochrome p-450 in aromatization. *J. Biol. Chem.*, **249**, 5373.

VERMEULEN, A. (1976). The hormonal activity of the postmenopausal ovary. *J. Clin. Endocrinol. Metab.*, **42**, 247.

VERMEULEN, A., PARIDAENS, R. & HEUSEN, J.C. (1983). Effects of aminoglutethimide on adrenal steroid excretion. *Clin. Endocrinol.*, **19**, 673.

VOLK, H., DEUPREE, R.H., GOLDENBERG, I.S., WILDE, R.C., CARABASI, R.A. & ESCHER, G.C. (1974). A dose response evaluation of delta-1-testololactone in advanced breast cancer. *Cancer*, **33**, 9.